# Comparison of the smiley face rod method versus intervertebral fusion for the treatment of L5 spondylolysis in adults

**DOI:** 10.3389/fmed.2024.1499773

**Published:** 2024-11-15

**Authors:** Qi-Yuan Dong, Xue-Fei Yang, Hai-Long Wu, Yan Shi, Li-Min Yu, Yong-Can Huang, Bin-Sheng Yu

**Affiliations:** ^1^Shenzhen Key Laboratory of Spine Surgery, Department of Spine Surgery, Peking University Shenzhen Hospital, Shenzhen, China; ^2^Shenzhen Engineering Laboratory of Orthopaedic Regenerative Technologies, National & Local Joint Engineering Research Center of Orthopaedic Biomaterials, Peking University Shenzhen Hospital, Shenzhen, China; ^3^Institute of Orthopaedics, Peking University Shenzhen Hospital, Shenzhen Peking University-Hong Kong University of Science and Technology Medical Center, Shenzhen, China

**Keywords:** smiley face rod, intervertebral fusion, spondylolysis, isthmic spondylolisthesis, isthmic repair

## Abstract

**Purpose:**

For patients who suffered from L5 spondylolysis and fail to improve using conservative treatment, the best surgical strategy remains controversial. This study compares the efficacy of the treatment of L5 spondylolysis using the smiley face rod (SFR) method versus intervertebral fusion (IF).

**Methods:**

We analyzed 38 patients with L5 spondylolysis who underwent surgery in our department between January 2017 and June 2019. Of these, 32 patients were included in our study: 14 patients in the SFR group and 18 patients in the IF group. The operation time, intraoperative blood loss, postoperative drainage time, length of stay and postoperative complications were compared. The pain visual analog scale (VAS) and Oswestry dysfunction index (ODI) were evaluated before operation and at 3 months, 6 months, and 1 year postoperatively. The changes in range of motion (ROM) in L4/5 and L5/S1 in these two groups before and after surgery were measured through imaging examinations and the bone graft fusion rate was assessed according to the Brown standard.

**Results:**

The operation time of the SFR group was much shorter than that of the IF group (98.8 ± 8.3 vs. 113.8 ± 8.6 min, *P* < 0.05), and the blood loss of the SFR group was significantly lower than that of the IF group (90.0 ± 43.9 vs. 175.0 ± 81.2 ml, *P* < 0.05). Length of stay in the SFR group was less than that of the IF group (9.5 ± 2.5 vs. 12.6 ± 3.2 d, *P* < 0.05). No difference was found in the VAS and ODI scores between the two groups at 3 months, 6 months, and 1 year after surgery. In the IF group, the ROM in L4/5 showed an obvious increase after surgery compared to that before surgery, and it was much bigger than that of the SFR group (*P* < 0.05). A notable reduction of ROM was seen in L5/S1 in the IF group compared to the SFR group (*P* < 0.05). The fusion rate of the isthmus in the SFR group was 79% at 3 months and 86% at 6 months after surgery. In the IF group, one patient suffered from adjacent segment degeneration (ASD), which caused compression symptoms in the lower extremity, and one patient suffered from an internal fixation fracture; these complications were not seen in the SFR group.

**Conclusion:**

The SFR and IF both improve the clinical symptoms and quality of life of patients with L5 spondylolysis. However, the SFR technique had the advantages of a shorter operation time and less blood loss than IF; it could also preserve the ROM of the surgical segment and had little influence on adjacent segments in short-term follow-ups.

## 1 Introduction

Lumbar spondylolysis refers to the discontinuation of the isthmus, with an incidence of 6% in the general population, and most patients are male (1, 2). It mostly occurs at L5 and in about half of patients with bilateral spondylolysis, and it leads to spondylolisthesis, as the defect of the posterior column exerts a greater load on the intervertebral disk (3, 4). Spondylolysis is a common cause of low back pain in adolescents, especially those who are closely related to repeated lumbar hyperextension and rotation (3, 4). The known pathogenesis of L5 spondylolysis is fatigue fracture caused by congenital weakness or defect in the lumbar isthmus due to long-term chronic injury (5). To date, conservative treatments are the gold standard treatment for L5 spondylolysis; the methods include physical therapy, lumbodorsal muscles exercises and block therapy (6, 7), which are able to improve the symptoms for most patients, especially adolescents. For patients who do not respond to conservative treatments, surgical interventions should be considered.

The major goal of surgery is to repair isthmus defects. Currently, intervertebral fusion (IF) is the commonest surgical procedure with the most affirmative effect, due to the firmest fixation (8–10); it has been widely used for patients of all age (11–13), especially for those with spondylolisthesis grade II and above or severe disk degeneration. Nevertheless, IF might result in a loss of motion, which further raises the risk of adjacent segment degeneration (ASD) (14, 15).

**FIGURE 1:**
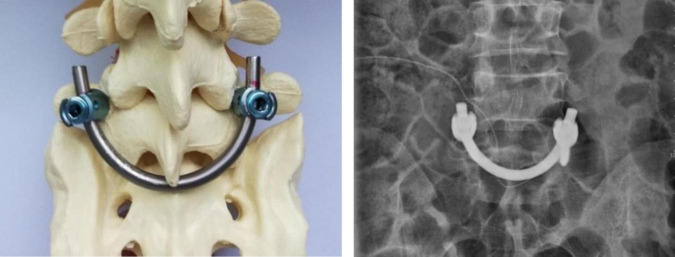
SFR shown on the model and X-ray. Two universal screws were inserted. A rod with a diameter of 5.5 mm was bent into a U-shaped rod and placed below the root of the spinous process of the L5. The rod end was connected and fixed with an ipsilateral pedicle screw. The screw holder was used to pressurize the broken end of the isthmus and then tighten the screw cap.

Direct repair has successful results for spondylolysis with or without low-grade spondylolisthesis (16). As one of the methods of direct repair, the smiley rod face (SFR, also known as the U-shaped rod) was first introduced by Altaf et al. (13, 17), and it has been used in the treatment of spondylolysis with or without grade I spondylolisthesis. As illustrated in Figure 1, the aim of this technique is to compress the defects using a U-shaped rod placed just caudal to the spinous process of the affected level, which is firmly fixed against the spinous process and lamina and promotes the compression of the graft in the defect. The satisfactory rate of SFR has been reported to range from 90 to 98% and its advantages are that it can preserve the motion of adjacent segments and prevent the degeneration of intervertebral disks (17–20). Nevertheless, some surgeons believe that the fusion rate of the SFR may be low due to insufficient fixation strengths (21, 22). However, most of the former reports are case series, and the efficacy and indications of this technique are still under debate.

Therefore, high-quality studies to compare the efficacy of SFR and IF are necessary. In this study, we retrospectively analyzed patients who received surgery for L5 spondylolysis using these two techniques, and we compared the efficacy, safety and complications. This work is meaningful for determining the safety and efficacy of SFR for L5 spondylolysis.

## 2 Materials and methods

### 2.1 Informed consent and ethics approval

This retrospective study was approved by the ethics committee of Peking University Shenzhen Hospital, with approval number 2022-034. Written informed consent was obtained from each patient before surgery.

### 2.2 Patients

We reviewed patients who were diagnosed with L5 spondylolysis at Peking University Shenzhen Hospital between January 2017 and June 2019. The inclusion criteria were as follows: (1) age < 40; (2) severe low back pain (VAS > 5) without symptoms and signs of nerve compression; (3) no symptom relief after 3 months of regular conservative treatments; (4) L5 spondylosis with or without spondylolisthesis and spondylolisthesis ≤ grade I; and (5) the Pfirrmann grade of the corresponding segmental intervertebral disks was ≤II grade. The exclusion criteria were (1) spondylolisthesis ≥ grade II; (2) disk herniation, spinal stenosis or other spinal disease; (3) the Pfirrmann grade of corresponding segmental intervertebral disks was ≥grade III; (4) patients with spinous process dysplasia or spinous process injury; and (5) patients who could not cooperate with postoperative follow-up for other reasons.

### 2.3 Data extraction

**FIGURE 2:**
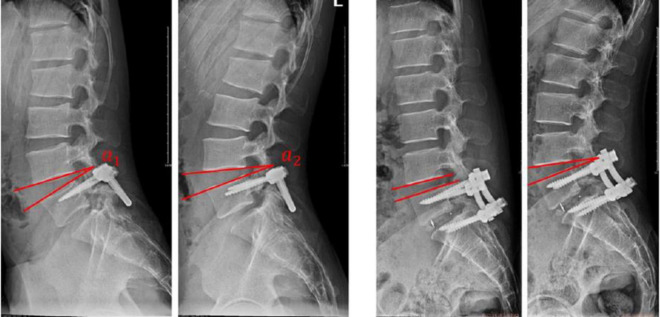
Measurement of the ROM. The angle between the lower edge of the proximal vertebral and the upper edge of the distal vertebrae in the flexion and extension positions of the lumbar spine was measured, and the difference between the angles was the ROM. **(a,b)** ROM(L4/5) = α_2_–α_1_, ROM(L5/S1) = α_4_–α_3_. **(c,d)** ROM(L4/5) = α_6_–α_5_.

The operation-related indexes, such as operation time, intraoperative blood loss, postoperative drainage time and lengths of stay, were recorded. The visual analog scale (VAS) and Oswestry disability index (ODI) before surgery and at 3, 6 months, and 1 year after surgery were collected to evaluate the clinical efficacy. ROM of L4/5, ROM of L5/S1 and global ROM of the lumbar vertebra were measured on X-ray images (Figure 2). Bone grafting fusion was defined in terms of three levels: (1) complete fusion, meaning that the space between the vertebral body and the implanted bone is completely bridged by trabeculae; (2) partial fusion, meaning that bridge trabeculae is less than 50%; and (3) non-fusion, which means that there is no trabecular bone in the interstitial space of the bone graft.

### 2.4 SFR fixation

After general anesthesia, the patients were placed in a prone position. A posterior midline incision about 4 to 6 cm was made with the L5 vertebral body at the center. The location of the isthmus defect was determined after the L5 spinous process, and lamina and superior articular processes were exposed layer by layer. The pedicle screw insertion points were determined according to the “herringbone crest” method, and two universal screws were inserted. The fibrous hyperplasia tissue near the isthmus was removed, and the bone of the broken end of the isthmus was resected until the blood oozed. A bone graft from the iliac bone was implanted into the isthmus defect. The bone and interspinous ligament were separated under the spinous process of L5, with a hole left. Then, a mold rod was bent to a U shape and connected to the pedicle screw at both ends, matching the width between the rod end and the screw tails. The bottom end of the rod passed through the hole. A rod with a diameter of 5.5 mm was bent into a U-shaped rod according to the shape of the mold rod and placed below the root of the spinous process of L5. The rod end was connected and fixed with an ipsilateral pedicle screw. The screw holder was used to pressurize the broken end of the isthmus and then tighten the screw cap. The wound was then washed, the negative pressure drainage tube was placed in the wound and the incision was sutured layer by layer.

### 2.5 IF

This study used the posterior lumbar IF technique. The lamina, articular and transverse processes of L5 and S1 were exposed in the same way as SFR. Four universal pedicle screws were placed in the L5 and S1, respectively. The nucleus pulposus, annular fibrous and cartilage plate of L5/S1 were removed, and an appropriately sized cage was taken and implanted into the intervertebral space. Autogenous iliac bone particles were implanted around the cage and compacted, and the caps were tightened after compression. The wound was then washed, the negative pressure drainage tube was placed in the wound and the incision was sutured layer by layer. Devices of both technique are from Weigao Shandong.

### 2.6 Statistical analysis

Statistical analyzes were performed using SPSS software (version 22.0; SPSS Inc., Chicago, IL, USA). Descriptive data are presented as mean ± standard deviation values. Categorical variables were tested with a χ2 test. Continuous variables were tested using an independent sample *t*-test. One-way ANOVA was used to analyze the follow-up data and imaging findings. A *P* < 0.05 was considered statistically significant.

## 3 Results

Of 38 patients who received surgical intervention in our department between January 2017 and June 2019, 32 met the inclusion criteria. Among them, 14 patients underwent SFR and 18 patients underwent IF. The average age of the patients was 28.7 ± 4.5 years (ranging from 23 to 36 years). All patients received spine X-ray, CT and MRI examinations. Table 1 shows the general information of the patients in each group. As shown in Table 2, the operation times and lengths of stay in the SFR group were significantly shorter than those in the IF group (98.8 ± 8.3 vs. 113.8 ± 8.6, *P* < 0.001); and the blood loss of the SFR group was significantly less than that of the IF group (90.0 ± 43.9 vs. 175.0 ± 81.2, *P* < 0.05).

**TABLE 1:** General information of patients in the two groups.

	SFR group (*n* = 14)	SSLIF group (*n* = 18)	*P*-value
Age (y)	28.7 ± 4.4	28.8 ± 4.7	0.965
Gender (M/F)	8/6	10/8	0.726
BMI (kg/m^2^)	22.1 ± 3.1	23.9 ± 4.8	0.289
Smoking (Y/N)	3/11	4/14	0.650
Spondylolisthesis (Y/N)	6/8	8/10	0.425
Conservative treatment time (m)	4.1 ± 0.9	4.4 ± 1.1	0.289
Follow-up time (m)	18.7 ± 4.5	19.2 ± 5.3	0.632

**TABLE 2:** Comparison of related indicators of operation and hospitalization between two groups.

	SFR group (*n* = 14)	SSLIF group (*n* = 18)	*P*-value
Operation time (min)	98.8 ± 8.3	113.8 ± 8.6	<0.001
Blood loss (ml)	90.0 ± 43.9	175.0 ± 81.2	0.004
Drainage time (d)	2.7 ± 1.1	2.8 ± 1.3	0.863
Lengths of stay (d)	9.5 ± 2.5	12.6 ± 3.2	<0.001

Before the operation, no significant difference was observed in the VAS scores between the two groups (5.7 ± 0.8 vs. 5.5 ± 0.8, *P* > 0.05); and the postoperative VAS scores of both groups were significantly lower than those before surgery. No significant difference was recorded in the VAS scores between the two groups at 3 months (3.2 ± 0.4 vs. 2.9 ± 0.5), 6 months (2.1 ± 0.7 vs. 1.9 ± 0.5), and 1 year (1.1 ± 0.5 vs. 0.8 ± 0.6) after operation. There was no significant difference in ODI scores between the two groups before operation and at 3 months, 6 months, and 1 year after operation. Table 3 shows the detailed scores and *P*-values of these two groups at different time slots.

**TABLE 3:** Comparison of VAS score, ODI between the two groups.

			SFR group (*n* = 14)	SSLIF group (*n* = 18)	*P*-value
VAS (score)	Before operation		5.7 ± 0.8	5.5 ± 0.8	0.610
	After operation	3 months	3.2 ± 0.4	2.9 ± 0.5	0.193
		6 months	2.1 ± 0.7	1.9 ± 0.5	0.501
		1 year	1.1 ± 0.5	0.8 ± 0.6	0.275
ODI (%)	Before operation		56.4 ± 5.6	52.1 ± 5.8	0.075
	After operation	3 months	25.8 ± 5.0	22.1 ± 5.8	0.111
		6 months	17.5 ± 3.0	17.4 ± 2.3	0.940
		1 year	9.8 ± 1.5	9.5 ± 1.4	0.581

In these two groups, the preoperative L4/5 ROMs of all the patients were quite similar (9.0 ± 1.2 vs. 8.8 ± 1.1, *P* > 0.05). In the SFR group, no notable change in the L4/5 ROM was recorded at 1 year postoperatively compared with that before surgery (9.0 ± 1.2 vs. 9.3 ± 1.4, *P* > 0.05). In the IF group, the ROM at 1 year after operation was much higher than the value before operation (8.8 ± 1.1 vs. 10.7 ± 1.1, *P* < 0.05) and it was much higher than that of the SFR group (10.7 ± 1.1 vs. 9.3 ± 1.4, *P* < 0.05). For the ROM of L5/S1, there was no difference between the two groups before operation and the values at all the follow-up points were greater than those before surgery in both groups. In the SFR group, the ROM of L5/S1 were retained about 54% at 3 months after operation, 69% at 6 months after operation, and about 80% at 1 year after operation, while in the IF group, ROM was totally lost due to the fusion procedure; hence, the ROM of L5/S1 after surgery was significantly different between the two groups (0 vs. 9.7 ± 1.8, *P* < 0.05) (Table 4).

**TABLE 4:** Comparison of ROM between the two groups.

			SFR group (*n* = 14)	SSLIF group (*n* = 18)	*P*-value
ROM of L4/5 (°)	Before operation		9.0 ± 1.2	8.8 ± 1.1	0.537
	After operation	1 year	9.3 ± 1.4	10.7 ± 1.1	0.012
ROM of L5/S1 (°)	Before operation		12.1 ± 2.9	12.0 ± 2.6	0.971
	After operation	1 year	9.7 ± 1.8	0	<0.001

**FIGURE 3:**
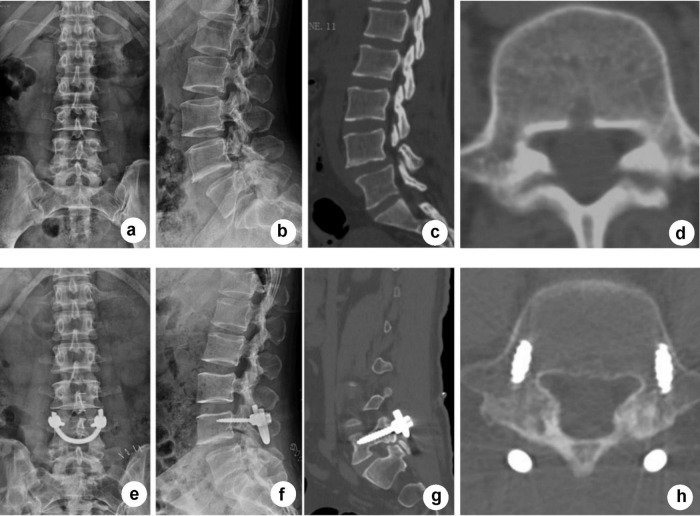
Typical case of SFR. A 37-year-old male patient who had suffered from low back pain for 4 years underwent SFR surgery. The pain was significantly reduced 1 year after surgery. **(a,b)** Anteroposterior and lateral X-ray CT before surgery showed L5 spondylolysis with grade I spondylolisthesis. **(c,d)** Sagittal and axial CT images before surgery showed L5 bilateral spondylolysis. **(e,f)** Anteroposterior and lateral X-ray after surgery showed that SFR provided strong fixation on the lesion. **(g,h)** CT showed that L5 spondylolysis reached preliminary fusion 1 year after surgery.

**FIGURE 4:**
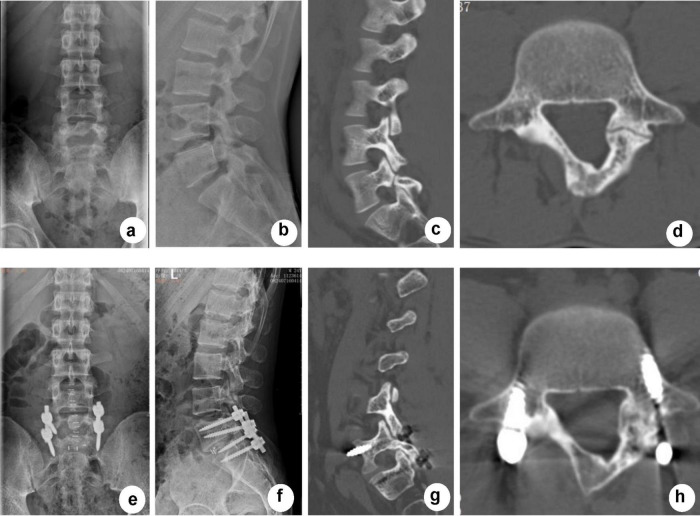
Typical case of SSLIF. A 35-year-old female patient who had suffered from low back pain for 3 years underwent SSLIF surgery. The pain was significantly reduced 1 year after surgery without ASD, screw loosening or rod fracture. **(a,b)** Anteroposterior and lateral X-ray CT before surgery showed L5 spondylolysis. **(c,d)** CT before surgery showed bilateral L5 spondylolysis. **(e,f)** Anteroposterior and lateral X-ray after surgery showed that SSLIF provided strong fixation and that the cage was in the proper position. **(g,h)** CT showed that pedicle screws in L5 and S1 were in position without loosening 1 year after surgery.

Isthmus fusion in the SFR group was evaluated by X-ray and CT at 1 year after surgery according to the Brown standard; non-fusion was seen in two patients and partial fusion was seen in the other two patients. The fusion rate of the SFR group was 86% in our study. In the IF group, one patient suffered ASD in L4/5 which further caused lower limb symptoms, and another patient suffered internal fixation fracture. During the follow-up period, no mechanical complications, such as rod fracture or screw looseness, occurred in the SFR group. Figures 3, 4 present typical cases for each group.

## 4 Discussion

Previous case series have reported that SFR could reach a satisfactory result for L5 spondylolysis, with a fusion rate of up to 98% (17–21, 23). Westacott and Cooke (22) compared the clinical efficacy of the internal fixation technique and the intersegmental fusion technique in the treatment of adolescent L5 spondylolysis, and the results showed that the long-term efficacy of the internal fixation group was worse than that of the intersegmental fusion group. Nevertheless, to date, few studies have been conducted to prove the clinical efficacy of SFR compared with IF. In this study, we first conducted a retrospective study to compare SFR and IF on several scales. The clinical efficacy of SFR showed no significant difference from IF, but SFR was able to minimize the wound of surgery, leading to less operation time, less blood loss and shorter lengths of stay. Additionally, with a 1-year follow-up, we preliminarily proved the advantage of SFR in preventing ASD, as we found that the SFR group reserved the ROM of L4/5 maximumly, and retained 80% of the ROM of L5/S1 at 1 year after operation.

The IF technique requires greater exposure of the vertebrae and the implantation of four pedicle screws, which would prolong the operation time and increase blood loss. Additionally, the larger operative wound may prolong the lengths of stay among the IF group.

The pedicle screw system combined with IF can provide effective support on the vertebral body and obtain better stability (11); a high rate of fusion and restoration of the physiological spine curvature, as well as early exercise, can be obtained. Thus, it has been widely used in the treatment of L5 spondylolysis. Nevertheless, the increase in ROM of the adjacent segment and change of motion pattern could lead to segmental degeneration (24). ASD does not always induce clinical symptoms (9), but the increased risk of ASD caused by cross-segmental internal fixation and intervertebral bone fusion remains a clinical problem (25). The SFR technique was able to directly repair and maintain the normal anatomical relationship, thus retaining the maximum ROM of the lumbar spine and avoiding ASD (26). Biomechanical experiments using human cadavers showed that SFR could improve the instability caused by L5 spondylolysis, increase the stability of the corresponding articular process (27) and prevent the degeneration of adjacent segments by reducing the load on the disks (14). Hence, compared with IF, SFR has clear advantages that not only provide local stability but also maintain the integrity of the spine. In this study, no ASD, fracture or loosening of internal fixation were detected in the SFR group, which suggests that SFR has a much lower risk of mechanical complications than the IF technique. However long-term follow-up should be conducted to get strong evidence.

When SFR was first applied to clinical practice, the non-fusion rate was up to 67% for patients with grade I spondylolisthesis; however, with the understanding of biomechanics and increasing practice, the fusion rate increased to 98% (17–19, 23). In our study, the fusion rate evaluated 1 year postoperatively in the SFR group was about 86% (12/14), which was consistent with previous studies. We believe that failure of the bone fusion might be related to the following: First, the insufficient elimination of isthmus fibrous tissue might block the fusion of the isthmus when the SFR technique was initially performed by surgeons. To decrease the high non-fusion rate of the isthmus, the fibrous tissue should be removed completely and bone grafting should be done if needed. Second, several studies have reported that the bone fusion rate was significantly higher in patients than in older ones (24, 28–30). Third, the obesity of patients might raise the mechanic load on the isthmus, leading to a failure of bone fusion. Spondylolysis accompanied by spondylolisthesis or other diseases might also be a risk factor for non-fusion.

Although SFR has the advantages we mentioned above, indications of SFR should be strictly defined. In our department, indications that we suggest include: ① young patients, especially younger than 30 years old; ② L5 spondylosis with or without spondylolisthesis and spondylolisthesis ≤ grade I; and (5) the Pfirrmann grade of the corresponding segmental intervertebral disks was ≤II grade.

We should acknowledge that there are some limitations to this study. First, this is a retrospective study and the sample size is small. High-level studies are needed to prove the superiority of SFR on L5 spondylolysis. Second, the follow-up period was not long enough to estimate the overall incidence of mechanical complications. Finally, biomechanical studies using cadavers and finite element analysis to understand the effects of these two techniques on the segmental and global kinematics of the spine are extremely necessary.

## 5 Conclusion

Both the SFR and IF were able to improve the clinical symptoms and quality of life of patients with L5 spondylolysis. The SFR technique had the advantages of shorter operation time and less blood loss than the IF technique. SFR could also preserve the ROM of the surgical segment and have little effect on adjacent segments in short-term follow-ups, leading to a lower risk of complications than IF.

## Data Availability

The raw data supporting the conclusions of this article will be made available by the authors, without undue reservation.
